# Nathan Carlin: *Pastoral aesthetics: a theological perspective on principlist bioethics*: Oxford University Press, New York, 2019, 216 pp, ISBN: 978-0-19-027014-8

**DOI:** 10.1007/s11017-020-09534-9

**Published:** 2021-02-12

**Authors:** Gaia De Vecchi

**Affiliations:** Catholic University of the Sacred Heart, Milan, Italy

It is well known that the term “bioethics” was first coined in the late 1960s by the oncologist Van Rensselaer Potter, who was looking for a field of knowledge to act as a bridge between philosophical and scientific research. One of the first challenges of bioethics was to endow itself with an epistemological statute. In 1979 Tom Beauchamp and James Childress published *Principles of Biomedical Ethics* [1], now a classic text in the bioethical literature. In the book, the two authors advance four ethical principles—which now act as a practical ethical paradigm—for the resolution of morally ambiguous cases which were, at that time, becoming increasingly frequent and varied in medicine. The four commonly accepted principles are as follows: respect for autonomy, beneficence, nonmaleficence, and justice. Over the years, Beauchamp and Childress’s principlist bioethics has been shown to have both strengths and weaknesses, as the authors themselves acknowledge [2].

Nathan Carlin, an ordained Presbyterian minister and Associate Professor in the McGovern Center for Humanities and Ethics at the University of Texas, develops an original reinterpretation of principlist bioethics built on Paul Tillich’s “method of correlation” [3]. Tillich’s method of correlation can be concisely characterized as a thought exercise whereby existential questions are correlated with answers drawn from Christian revelation. In this way, it is a philosophical reflection that the theologian (the pastor, the minister, the believer) develops in relation to her or his own faith.

In view of this method, starting from the four principles of medical ethics, Carlin argues for the cultivation of an aesthetic sensibility in bioethical reflection. Such a sensibility must be theologically informed, psychologically sophisticated, therapeutically oriented, and experientially grounded. There are a wide variety of resources available to achieve this goal: painting, fiction, memoir, poetry, journalism, cultural studies, clinical journals, and classic cases in bioethics, as well as some original pastoral care conversations. Nonetheless, the author emphasizes that throughout the book bioethics and theology—as poles of reflection—are to be understood according to a tension between opposing forces: “the four principles of bioethics may be viewed as centric forces in bioethics and … pastoral theology may be viewed as an eccentric force,” or a force “looking *at* or *from* the margins” (p. 31).

The book allocates each of the four principles its own chapter and organizes each of these chapters into a unified structure, allowing the reader to scan through and study the text in a simple and efficient way. To that effect, the author first discusses the bioethics principle at issue and offers one or two illustrations of the principle; he then discusses the image of pastoral care and then correlates this with the initial bioethics principle. The method, which is plainly interdisciplinary, in a certain sense echoes the words of Pope Francis in *Laudato Si’*:

Given the complexity of the ecological crisis and its multiple causes, we need to realize that the solutions will not emerge from just one way of interpreting and transforming reality. Respect must also be shown for the various cultural riches of different peoples, their art and poetry, their interior life and spirituality. If we are truly concerned to develop an ecology capable of remedying the damage we have done, no branch of the sciences and no form of wisdom can be left out, and that includes religion and the language particular to it. The Catholic Church is open to dialogue with philosophical thought; this has enabled her to produce various syntheses between faith and reason. [4, §63].

In this path of correlation, the four principles are read in such a way as to show how theology (particularly pastoral theology) can and should revitalize bioethical reflection. Although Carlin himself hails from a liberal Protestant theological perspective, he declares that his interest is directed more toward the pastoral sphere: “the perspective offered in this book is less denominational than pastoral” (p. 144). Briefly put, in Carlin’s words, the contribution that pastoral theology can offer to principlist bioethics is as follows (p. 144):

1. a pastoral perspective on respect for autonomy shifts the focus away from a respect of choice to an appreciation of individuals;
2. a pastoral perspective on nonmaleficence considers harm not only in light of physical and psychosocial considerations but also in light of idolatry;
3. a pastoral perspective on beneficence places an emphasis on existential well-being as influenced by the caregiver; and
4. a pastoral perspective on justice requires attention to be given to the local manifestations of systemic sin by listening to the voices of oppressed individuals.

Finally, it is worth noting that Carlin’s text, which is not meant to be a how-to book on bioethics but a clear and productive way of looking at bioethical questions, also offers its readers a wide, interesting and up-to-date bibliography.
